# Align Appalachia’s Opioid-Settlement Spending with Evidence and Emerging Threats

**DOI:** 10.13023/jah.0703.01

**Published:** 2025-09-01

**Authors:** Bradley Firchow

**Affiliations:** University of Kentucky

**Keywords:** Appalachia, opioid settlement, synthetic drugs

## Abstract

State and local governments across Appalachia are allocating opioid-settlement dollars over the coming years. This funding opportunity can support lasting public health infrastructure or be spent on short-term programs anchored in the opioid crisis of the past. Evidence indicates that emerging synthetic drugs such as nitazenes and xylazine are altering overdose risk patterns in ways that require urgent policy attention.

State and local governments across Appalachia are allocating opioid-settlement dollars over the coming years. This funding opportunity can support lasting public health infrastructure or be spent on short-term programs anchored in the opioid crisis of the past. Evidence indicates that emerging synthetic drugs such as nitazenes and xylazine are altering overdose risk patterns in ways that require urgent policy attention. Nitazenes can be up to 40 times more potent than fentanyl, and xylazine is involved in approximately 30% of overdose deaths in some rural counties.^1^,^2^,^3^

Nitazene-class opioids have reappeared in illicit drug supplies with extreme potency. Laboratory analyses report that isotonitazene exhibits potency far greater than fentanyl.^1^ Nitazenes, a potent class of illicit synthetic opioids derived from 2-benzylbenzimidazole, have been increasingly identified in toxicology reports and death certificates. However, they are often missed by standard laboratory panels, a limitation that is especially consequential for rural emergency departments with restricted access to advanced testing.^4^ Xylazine is an α2-adrenoceptor agonist veterinary sedative not approved for human use; its involvement in overdoses has risen rapidly across North America, spreading from early Northeastern epicenters into Southern and Western regions and frequently co-occurring with illicitly manufactured fentanyl in a polysubstance ‘syndemic.’^2^,^5^

Four policy principles, supported by recent research, should guide settlement spending. First, investments must be paired with explicit evaluation frameworks and public reporting. ASPPH Task Force on Opioid Use Disorder Prevention and Treatment concludes that maximizing settlement benefits requires transparency, equity-focused disbursement, and outcome monitoring.^6^ Adaptive funding mechanisms that shift resources based on evolving needs are essential in the Appalachian context.

Second, surveillance systems must expand beyond mortality counts to incorporate near-real-time supply data. Drug-checking technologies deployed through syringe service programs have detected xylazine and other adulterants commonly missed by traditional toxicology methods.^7^ Fentanyl test strip studies demonstrate that individuals modify their drug use behaviors when they know fentanyl is present, reducing exposure risk.^8^ Settlement funds can finance portable spectrometry, laboratory partnerships, and standardized inter-county reporting.

Third, clinical capacity should be strengthened for synthetic exposures. Because xylazine is not an opioid, naloxone does not counteract its pharmacologic effects; presentations often include prolonged sedation and severe soft-tissue injury, underscoring the need for enhanced drug checking, surveillance, and harm-reduction services rather than opioid-only protocols.^5^ Emergency providers need to manage sedation and cardiovascular effects that differ from opioid toxicity. Rural EMS and clinics should receive protocol updates, simulation-based training, and wound-care supplies, along with strengthened referral pathways for addiction treatment.

Fourth, accountability mechanisms must reflect regional disparities. In 2021, adults 25 – 54 in Appalachian counties had overdose mortality levels nearly 72% greater than the rest of the United States.^9^ Allocation formulas based solely on population risk underfunding the communities with the greatest burden. County-level dashboards showing spending, program reach, overdose trends, and clinical outcomes can improve transparency and support cross-jurisdiction learning.

Based on these principles, a practical research and policy agenda should include:

1. Developing standardized evaluation metrics and quasi-experimental designs, such as interrupted time series, to assess program impact, with independent review as a funding condition.^6^
2. Piloting and validating mobile drug-checking models in rural environments, integrated with public-health and medical examiner data.^7^
3. Convening expert panels to develop consensus guidelines for nitazene and xylazine management tailored to low-resource settings.^5^
4. Mapping settlement spending against epidemiologic data to identify under-resourced areas, with mandated community representation in decision-making.^6^

The legacy of this funding will be defined by whether Appalachia builds adaptable, data-driven public-health systems. Policymakers, health agencies, and community organizations can use settlement dollars to implement robust evaluation, invest in surveillance, and prepare clinicians for emerging synthetic threats. These actions will not only address the current crisis but also strengthen resilience against future public-health challenges.
